# Unraveling of Molecular Mechanisms of Cognitive Frailty in Chronic Kidney Disease: How Exercise Makes a Difference

**DOI:** 10.3390/jcm13195698

**Published:** 2024-09-25

**Authors:** Vasiliki Michou, Georgios Tsamos, Dimitra Vasdeki, Asterios Deligiannis, Evangelia Kouidi

**Affiliations:** 1 Sports Medicine Laboratory, School of Physical Education & Sport Science, Aristotle University, 57 001 Thessaloniki, Greece; adeligia@phed.auth.gr (A.D.); kouidi@phed.auth.gr (E.K.); 2 Division of Endocrinology and Metabolism and Diabetes Centre, First Department of Internal Medicine, Medical School, AHEPA University Hospital, Aristotle University of Thessaloniki, 546 36 Thessaloniki, Greece; tsamgeor@gmail.com (G.T.); demivs14@gmail.com (D.V.)

**Keywords:** chronic kidney disease, cognitive impairment, frailty, mechanisms, exercise, physical activity

## Abstract

As our population ages, the medical challenges it faces become increasingly acute, with chronic kidney disease (CKD) becoming more prevalent among older adults. Frailty is alarmingly more common in CKD patients than in the general populace, putting the elderly at high risk of both physical and cognitive decline. CKD not only accelerates physical deterioration, but also heightens vascular dysfunction, calcification, arterial rigidity, systemic inflammation, oxidative stress, and cognitive impairment. Cognitive frailty, a distinct syndrome marked by cognitive deficits caused by physiological causes (excluding Alzheimer’s and other dementias), is a critical concern. Although cognitive impairment has been well-studied, the molecular mechanisms driving cognitive frailty remain largely uncharted. Comprehensive interventions, including cutting-edge pharmaceuticals and lifestyle changes, are pivotal and effective, especially in the early stages of CKD. Recent research suggests that systematic exercise could counteract cognitive decline by improving brain blood flow, boosting neuroplasticity through the brain-derived neurotrophic factor (BDNF), and by triggering the release of neurotrophic factors such as insulin-like growth factor (IGF-1). This review delves into the molecular pathways of cognitive frailty in CKD, identifies key risk factors, and highlights therapeutic approaches, particularly the potent role of exercise in enhancing cognitive health.

## 1. Introduction

Chronic kidney disease (CKD) is increasingly prevalent worldwide, affecting approximately 850 million people among the global population [1]. It causes over 1.2 million deaths and results in the loss of 28 million years of life annually [2]. Despite heightened awareness, CKD’s burden continues to escalate, particularly in low-income regions. Extensive research has been conducted in recent years on cognitive performance across all stages of CKD [3,4,5,6,7,8]. Cognitive impairment is highly prevalent among CKD patients, adversely affecting their outcomes. Patients with cognitive impairment exhibit a lower quality of life (QoL), higher mortality rates during dialysis, and increased healthcare utilization and hospitalization days [9,10,11]. Cognitive deficits are observed in most areas, notably impacting executive functions [12]. Therapeutic options for cognitive impairment associated with CKD remain exceedingly limited.

Frailty, characterized by increased vulnerability and reduced functionality due to age or disease-related damage to bodily systems, results in a diminished ability to respond to stressors and poses substantial challenges, especially for those with chronic illnesses and the elderly [13,14]. Cognitive frailty, a distinct subset of frailty, encapsulates both physical frailty and cognitive deficits. Despite its significance within populations afflicted by chronic conditions and the elderly, the evaluation of cognitive function remains conspicuously absent from established frailty assessment paradigms, such as the frailty phenotype or frailty index which were established in 2001 and 2006, respectively [15,16]. However, in 2013, the International Academy on Nutrition and Aging (IANA) and the International Association of Gerontology and Geriatrics (IAGG) defined ‘cognitive frailty’ as a unique clinical manifestation involving both physical frailty and cognitive impairment, as determined by a clinical dementia rating (CDR) score of ≥0.5, while excluding Alzheimer’s and other dementias [17]. Cognitive impairment, encompassing deficits in learning, memory, and sensory processing, commonly accompanies CKD, with prevalence rates ranging from 16% to 38% across different disease stages [18]. Research indicates a close association among physical vulnerability, sarcopenia, and cognitive impairment, underpinned by mitochondrial derangement, epigenetic modulations, and oxidative stressors, with deleterious repercussions on metabolic equilibrium, stress responsiveness, and neuromuscular integrity [19].

Recently, the association between frailty and cognitive impairment has been documented in non-dialysis, hemodialysis (HD), peritoneal dialysis patients, and kidney transplant recipients [20,21,22,23,24]. CKD patients, due to factors like anemia, inflammatory vasculopathy, and metabolic disorders, demonstrate heightened susceptibility to cognitive impairment, with prevalence rates varying from 10% to 40% [11,25,26,27,28,29,30,31] (Table 1). Among CKD patients, those undergoing HD for end-stage disease emerge as bearing the greatest burden of cognitive impairment. It is imperative for healthcare providers to discern this vulnerable subset and dedicate concerted efforts to the amelioration of modifiable risk factors.

Furthermore, a crucial aspect of frailty is physical inactivity, suggesting that interventions to increase physical activity levels may directly or indirectly reverse frailty (both physical and cognitive). Therefore, the role of exercise in managing frailty is significant, as it can enhance physical performance, functional capacity and mental health, reduce the risk of sarcopenia, and alleviate symptoms of fatigue, exhaustion, and cognitive dysfunction [34]. Regular exercise is considered a cornerstone of the management of CKD. Interventional exercise programs are beneficial, feasible, and safe for CKD patients undergoing hemodialysis [35]. However, few clinical and retrospective studies regarding the exercise of patients with ESRD have proved that increasing the level of physical activity or maintaining moderate-to-high levels of physical activity long-term may benefit the physical and mental health of this vulnerable group of patients.

This review undertakes a comprehensive exploration of the intricate molecular pathways underpinning cognitive frailty within the context of CKD. It meticulously identifies pivotal risk factors contributing to this multifaceted condition and highlights therapeutic modalities, with a particular emphasis on the formidable potential of exercise with respect to bolstering cognitive well-being.

## 2. Mechanisms Underlying the Pathogenesis of Cognitive Frailty in CKD

There are two CKD-related mechanisms underlying the pathogenesis of mild-to-severe cognitive impairment: the vascular and neurodegenerative hypotheses. The vascular hypothesis is based on the high prevalence of cardiovascular disease (CVD) risk factors (i.e., hypertension, diabetes mellitus, and dyslipidemia) and on the significant burden of both symptomatic (stroke and transient ischemic attack) and subclinical cerebrovascular diseases (small-vessel infarcts, lacunes, and white matter disease) [36,37]. The cognitive impairments linked to cerebrovascular disease mainly impact processing and executive functions. These cognitive areas involve planning and task execution. The majority of studies show that individuals with CKD are most affected in terms of processing speed and executive function [12,32,38], while CVD and its risk factors negatively impact cognitive function. In the early stages of CKD, the presence of albuminuria, indicative of systemic vascular injury, is linked to worse cognitive function and to the development of dementia [37,38,39]. Additionally, as CKD progresses and kidney function declines, cognitive performance steadily worsens. In contrast, cognitive impairment shows favorable improvements after kidney transplantation. Longitudinal studies show improved cognitive function 6-to-12 months after transplantation, with continued improvement in subsequent years, potentially affected by frailty [5,20,40]. These findings have two potential explanations: first, successful kidney transplantation restores crucial functions essential for optimal cognitive function, and, second, transplantation eliminates the need for dialysis and associated complications that may promote cognitive impairment [41]. Based on the above elements, cerebrovascular disease is unlikely to be the only contributing factor to cognitive impairment in individuals with CKD [5,40].

Furthermore, according to the neurodegenerative hypothesis, the build-up of uremic toxins can lead to cerebral endothelial dysfunction, potentially contributing to cognitive decline [42]. Uremic toxins, which are byproducts of CKD, can breach the blood–brain barrier, a protective membrane that normally prevents harmful substances from entering the brain. Once inside, these toxins can cause cognitive dysfunction and neurodegeneration. Notably, toxins that have been linked to an increased risk of cognitive impairment in CKD patients include phosphate, para-cresyl sulfate (PCS), indoxyl sulfate (IS), and fibroblast growth factor 23 (FGF23) [43], as well as neuropeptide Y, a polypeptide implicated in some neurodegenerative and neuroimmune disorders [44] and present in high levels in CKD patients [45]. Yeh et al. [43], in a study that included 199 CKD patients and 84 control subjects, found that patients with higher serum PCS and IS levels had poorer cognitive function in the early stage of CKD. Moreover, the study indicates that neuronal damage induced by uremic toxins may be more critical than disturbed hemodynamic factors or lipid metabolism in cognitive impairment pathogenesis. Experimental models have also shown that the brain monoaminergic system is susceptible to uremic neurotoxins [46].

However, this dichotomous perspective of potential pathogenesis for CKD-related neurocognitive disorders is likely an oversimplification of a multifactorial process that includes elements of both hypotheses [47].

## 3. Risk Factors

**Figure 1:**
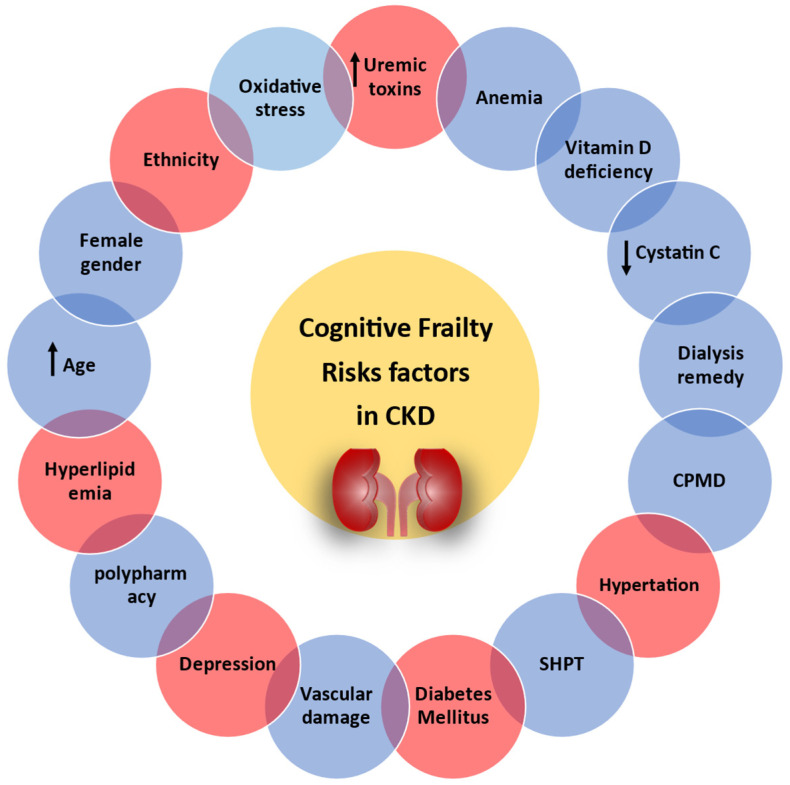
Traditional and non-traditional risk factors for cognitive frailty in CKD patients. Red color = traditional risk factors. Blue color = non-traditional risk factors. ↑ = increase and ↓ = decrease. CPMD: calcium and phosphorus metabolism disorder; SHPT: secondary hyperparathyroidism.

Among the most frequently reported risk factors for cognitive frailty in CKD patients are age, the female gender [6], HD remedy, vascular damage, uremic toxicity, inflammation, oxidative stress, diabetes mellitus, cardiovascular disease [6,10,18], and others [48] (Figure 1).

### 3.1. Age, Oxidative Stress, and Inflammation

In general, brain aging, with its unique molecular and structural complexity, shares many cellular and molecular aspects with other organ systems (e.g., oxidative stress, mitochondrial dysfunction, dysfunctional protein homeostasis, etc.). Nevertheless, there are age-related changes that are distinctly observed in the brain, adding to its intriguing nature. Among the most prominent structural changes caused by an aging brain are increased number of activated astrocytes and microglia, reduced neurogenesis, reduced white and gray matter volume, increased cerebrospinal fluid, volume alterations and changes in mitochondria’s morphology, as well as dysfunction of the hippocampus [49]. Several studies suggest that cognitive frailty is a significant concern in elderly HD patients, with a prevalence ranging from 4.6% to 25.9% [22,50]. However, the research landscape is still sparse, with few studies and limited evidence on cognitive frailty in elderly patients with CKD. The reported prevalence varies greatly due to differences in the population studied and the measurement tools used, highlighting the need for more comprehensive research in this area [15].

Furthermore, as CKD progresses, patients not only experience chronic inflammation (accompanied by higher levels of CRP, IL-6, and TNF-α) and higher uremic toxins levels [51], but also a substantial increase in plasma oxidative stress markers [such as reactive oxygen species (ROS) and malondialdehyde (MDA)]. This increase is particularly pronounced in end-stage renal disease (ESRD) patients undergoing HD. Oxidative stress molecules contribute to progressive kidney damage by promoting renal ischemia, glomerular damage, cell death, and apoptosis, eventually stimulating a severe inflammatory process [52]. Increased oxidative stress also contributes to cognitive frailty accompanying aging. The brain is especially prone to the damaging effects of oxidative damage because it has low levels of free radicals and protective antioxidants, uses a lot of oxygen, contains high levels of iron and easily peroxidizable fatty acids, and because nervous tissue is essentially non-regenerative [53].

### 3.2. Female Gender

Regarding cognitive vulnerability and CKD, at the level of gender, women with advanced CKD appear to experience significant effects on cognitive and executive function, language, and memory, resulting in reduced psychological well-being [54]. The cognitive decline seen in women is likely related to GFR levels. Kong et al. [55], studying the correlation between renal and neurocognitive function in women over 70, observed a strong correlation between immediate and short-term memory and renal function in women. Similarly, in the study by Sajjad et al. [54], having mild renal impairment was found to be associated with a faster cognitive decline in women compared to men. A possible etiology for the reduced cognitive function experienced by women is the depletion of estrogen during the postmenopausal period. However, the effect of estrogen-containing hormonal regulators on enhancing cognition or reducing the risk of cognitive decline is controversial, and the results are mainly inconsistent [56]. Evidence for gender-specific CKD effects exists for Alzheimer’s disease, indicating almost twice the incidence of cognitive decline in women compared to men. Although Alzheimer’s disease is not included in the definition of cognitive vulnerability, the cognitive impairment that characterizes both clinical conditions in women with CKD can be considered a significant finding [57].

### 3.3. HD Remedy

CKD patients undergoing the HD maintenance remedy are at high risk of cognitive vulnerability, while the method of extrarenal dialysis appears to be directly related to the progression of cognitive decline. In contrast to peritoneal dialysis, HD causes frequent and significant blood pressure fluctuations, leading to cardiovascular and cerebral implications. Notably, mean blood flow velocity (the primary measure of cerebral blood flow) is significantly reduced during HD. While induced intradialytic hypotension has been shown to strongly associate with cerebral atrophy in CKD patients [58], cerebral atrophy is often seen in HD patients under 50 years old, even without a history of stroke. It is directly linked to the blood pressure measured before the start of the dialysis session, the patient’s history of high blood pressure, and the number of years they have been undergoing dialysis [58,59,60]. According to Mizumasa et al. [58], ischemia is considered a possible factor in the induction of cerebral atrophy in HD patients. These findings collectively suggest that hypotensive episodes during or immediately after the dialysis session can cause ischemic brain damage due to the ensuing hemodynamic changes. Repeated exposure to these events can eventually result in the loss of nerve cells and fibers, leading to further progression of brain atrophy. Additionally, the hemodynamic instability observed during HD sessions has been linked to subsequent brain damage and cerebral microbleeds, serving as an additional risk factor for cognitive impairment [47].

It is important to consider the influence of the temperature and rate of ultrafiltration of the dialysate on cerebral blood flow, as it can significantly impact cognitive function [61]. The impact of the dialysis dose on cognitive function, particularly with increased dialysis dose and flux, remains uncertain. A study that examined the connection between dialysis adequacy and cognitive function in a group of patients undergoing HD maintenance found no link between a lower Kt/V value and poorer cognitive performance [62]. This underscores the need for future studies to investigate the long-term relationship between dialysis adequacy and cognitive function to verify these results.

### 3.4. Secondary Hyperparathyroidism

Secondary hyperparathyroidism (SHPT), characterized by severely elevated parathyroid hormone levels (PTH), is a common metabolic abnormality and a potentially modifiable dementia risk factor in ESRD patients. PTH can cross the blood–brain barrier, where it can bind to receptors and affect cognition [63,64]. It is crucial to follow treatment guidelines for SHPT, as several single-institution studies have demonstrated improvement in specific cognitive domains after treatment, which includes medications like phosphate binders, vitamin D analogs, and calcimimetics or surgical parathyroidectomy [65,66,67,68]. Controlling SHPT with calcimimetics may improve cognitive function by preventing increased calcium levels in the brain, potentially interfering with neurotransmission [69]. Moreover, vitamin D wields neuroprotective and regulatory roles in the central nervous system, and its deficiency is common in CKD patients. Based on this, vitamin analogs, such as calcitriol, a vitamin D receptor activator (VDRA), are highly important in SHPT remedies for CKD patients [70]. However, it is concerning that, despite these guidelines, SHPT remains untreated in up to 30–60% of patients. This represents a significant missed opportunity to address one of the central complications of ESRD: cognitive decline and dementia [65,68,71]. In brief, a profound comprehension of the molecular mechanisms of the FGF23/α klotho axis is crucial to understand cognitive decline and develop new therapeutic approaches for CKD patients.

### 3.5. Anemia

Elderly patients with CKD are often at higher risk of experiencing cognitive frailty due to anemia, malnutrition, inflammatory vascular diseases, and various metabolic disorders [72]. Anemia is common in patients with CKD. Kurella-Tamura et al. [73] investigated the relationship between anemia and cognitive decline in 762 adults aged ≥ 55 years who were affected by CKD and found that 46% of them had anemia. They also noticed that, among older adults with CKD, anemia was not independently associated with baseline cognitive function or decline. Koyama et al. [74], by conducting a retrospective cohort study involving 620,095 veterans aged 45 and older with incident stage 3 CKD, found a significant association between anemia and an increased risk of dementia among veterans with incident CKD and concluded that anemia is a possible predictor of dementia risk. Similarly, according to the results from the National Health and Nutrition Examination Survey by Blasco-Colmenares et al. [75], anemia is significantly correlated with impaired cognitive function domains, such as impaired executive function, impaired processing speed, and sustained attention, in CKD patients (disease stage 3–5). It has also been shown that hemoglobin (Hb) values between 11.5 g/dL and 12.5 g/dL are associated with cognitive function improvement and increased blood flow in the middle cerebral artery via transcranial Doppler testing [76]. Thus, clinical management of anemia may be of high importance to enhance cognitive function in patients with CKD.

### 3.6. Calcium and Phosphorus Metabolism Disorder

Calcium and phosphorus metabolism disorder (CPMD) is a common complication in CKD patients. Calcium–phosphate deposits in the soft tissues can elevate the risk of CVD and cerebrovascular issues in patients with HD [77]. Higher levels of phosphorus in HD patients leads to hyperphosphatemia, which increases the risk of brain hemorrhage [78]. Conversely, hypercalcemia leads to elevated brain calcium levels, resulting in increased neuronal excitability [79]. In addition, CPMD is accompanied by increased levels of FGF23 and PTH and decreased levels of α-Klotho and calcitriol, consequently leading to cerebrovascular diseases and related cognitive impairment [80,81]. Drew et al. [82] demonstrated a correlation between elevated levels of FGF23 and poor performance on comprehensive memory scores in 263 CKD patients undergoing HD maintenance [82]. The expression of the α-Klotho was highest in the kidney and parathyroid gland, followed by the brain. Research using magnetic resonance imaging (MRI) has also shown that lower levels of circulating α-Klotho are associated with increased risk of dementia and deep white matter lesions in the brain [83], as well as with the risk of B2 microglobulin-related amyloidosis and dementia [84].

In addition, recent research data suggest an association between CKD mineral and bone disorder and anemia. More precisely, it has been found that high serum phosphate levels are associated with low Hb levels in CKD patients who are not receiving treatment for anemia [85]. Anemia is considered a risk factor in the development of CVD and vice versa. Higher phosphate levels promote vascular calcification and further increase the risk of CVD. Therefore, it cannot be ruled out that phosphate levels may be linked to anemia due to increased arteriosclerosis and CVD risk [85]. However, in the KNOW-CKD study by Ryu et al. [86], calcium and phosphorus concentrations were identified as independent risk factors for anemia, highlighting the need for further investigation into these associations [87]. Meanwhile, clinical studies in CKD patients have revealed that higher serum FGF23 levels are negatively associated with lower serum Hb levels [88]. Nam et al. [89], in a retrospective cohort study of 2089 patients with non-dialysis CKD, revealed that higher FGF23 levels are correlated with an increased risk of anemia, while Mehta et al. [90], in a prospective cohort study of 3869 individuals with mild to severe CKD, also noticed that higher levels of baseline FGF23 were strongly and monotonically associated with prevalent anemia. However, these studies involved patients who were already undergoing treatment for anemia and did not reveal a clear association unaffected by the treatment. Based on the studies above, CKD mineral and bone disorders and anemia are closely associated with the cognitive impairment often observed in CKD patients.

## 4. Therapeutic Interventions: How Does Exercise Makes a Difference?

Despite the limited ways to slow the progression of kidney disease, research has shown that frailty can be reversed, particularly in its early stages. The primary goal of the global medical community is to prevent or delay functional decline and disability, which is characterized by a loss of personal self-sufficiency. There are no established treatments to prevent cognitive decline in individuals with CKD, and the limited range of medications available for cognitive impairment yields only modest effects [91]. However, pharmacological interventions targeting the mechanisms mentioned above and risk factors hold great promise for addressing cognitive frailty in CKD patients, offering a hopeful outlook for the future of patient care. Among them are anti-vascular calcification agents, anti-inflammatory agents, inhibitors of the renin–angiotensin system (RAS), anti-anemia drugs, vitamin D analogs, anti-diabetic drugs, anti-aging protein klotho, and others [92]. There is an urgent and critical need to evaluate therapies to forestall cognitive impairment and maintain or improve cognitive function, especially in older adults with CKD. This task is of the utmost importance and should be a top priority in the field of medical research. 

But what about the non-pharmacological effects of systematic exercise on cognitive frailty in this vulnerable population of CKD patients? The mechanisms behind exercise-induced improvements in neuronal health, synaptic function, neurogenesis, and cognitive function are complex and multifaceted. They involve a variety of processes such as increased blood flow to the brain, enhanced neuroplasticity, and the release of transcription and neurotrophic factors. These mechanisms are currently under investigation. To date, few investigations have been conducted into the role that exercise training may play in the brain structure and function of chronically ill patients such as CKD patients. Cai et al. [93], in a meta-analysis and systematic review of randomized controlled trials regarding the effects of exercise on cognitive function in patients suffering from a chronic disease, observed that low-intensity and moderate-intensity exercise interventions improve cognitive function in patients affected by chronic diseases. Physical activity and fitness levels are likely related to CKD patients’ cognitive function, but findings are inconclusive (Table 2).

**Figure 2:**
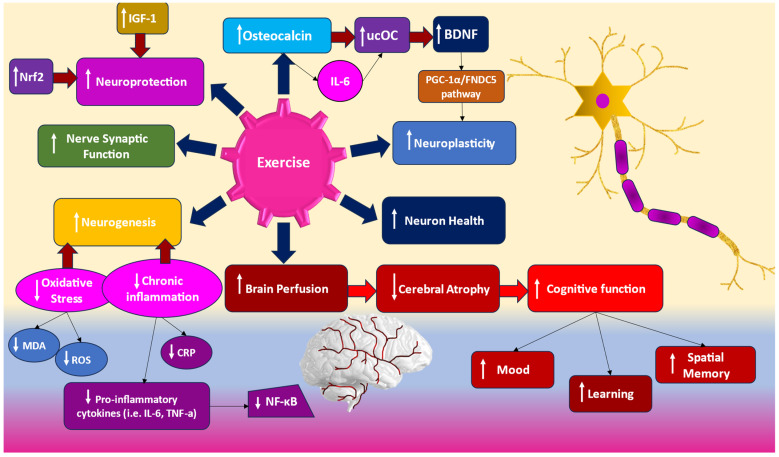
Exercise-induced mechanisms contrasting cognitive decline in subjects with or without CKD. ↑ = increase and ↓ = decrease. Nrf2: nuclear factor erythroid 2-related factor 2; NF-κΒ: nuclear factor κappa B; TNF-α: tumor necrosis factor-alpha; IL-6: interleukin 6; ROS: reactive oxygen species; MDA: malondialdehyde; BDNF: brain-derived neurotrophic factor; IGF-1: insulin-like growth factor; ucOC: uncarboxylated osteocalcin; PGC-1a: peroxisome proliferator-activated receptor γ coactivator 1α; FNDC5: fibronectin type III domain-containing 5.

Exercise training profoundly impacts the vascular system in CKD patients by reducing systematic inflammation, oxidative stress, and arterial stiffness, leading to an improved vascular milieu. Recent studies suggest that physical exercise could reduce plasma levels of oxidative stress biomarkers and improve the antioxidant defense system in CKD patients undergoing hemodialysis [101]. According to a recent systematic review and meta-analysis by Bradshaw et al. [102], results showed that, across the spectrum of CKD, exercise had a small but statistically significant effect on cognitive function, with aerobic exercise being particularly beneficial. Long-term aerobic exercise has been shown to reduce markers of oxidative stress, such as F2-isoprostanes and MDA, and improve antioxidant capacity by increasing levels of catalase and superoxide dismutase (SOD) [45]. Regular aerobic exercise can also reduce the levels of C-reactive proteins (CRPs), interleukin 6 (IL-6), tumor necrosis factor-alpha (TNF-α), soluble tumor necrosis factor receptor-1 (sTNFR1), and soluble tumor necrosis factor receptor-2 (sTNFR2), while promoting the production of anti-inflammatory factors such as interleukin 10 (IL-10), IL-1 receptor antagonist (IL-1RA), interleukin 4 (IL-4), and transforming growth factor beta-1 (TGF-β1) [103,104]. Considering that oxidative stress and nervous system inflammation contribute to neurodegeneration [105], oxidative stress has been demonstrated to be a critical factor in ageing-related neurodegenerative diseases [92], and, given the fact that systematic exercise may be one of the crucial ways to regulate oxidative and inflammatory responses in Alzheimer’s’ disease [106], there is a compelling reason to be optimistic about the role of exercise in regulating cognitive frailty in CKD patients. Figure 2 represents exercise-induced effects on neurons and the brain.

Additionally, considering the effects of exercise on the nuclear factor erythroid 2-related factor 2 (Nrf2) activation and nuclear factor κappa B (NF-κΒ) expression, previous studies have shown that Nrf2 is necessary for redox adaptations to exercise and as a mediator of the protection promoted by physical activity against ROS-induced muscle or other tissue damage. In recent years, attention has also turned to Nrf2’s role in the brain and to the different neurological diseases such Alzheimer’s and Parkinson’s [107]. Its activation can elicit neuroprotection and lead to cognitive enhancement [108,109]. This is particularly significant in the context of CKD, where oxidative stress is prevalent and can lead to cognitive dysfunction. Until recently, there were no exercise intervention studies on Nrf2 and NF-κΒ expression in CKD patients, but only studies in rats. Abreu et al. [110] were the first to investigate the effects of a 3-month resistance training regime on Nrf2 and NF-κΒ modulation in HD patients. They showed an upregulation of Nrf2 expression, but no significant changes were observed in NF-B expression [25]. Similarly, Brito et al. [111], by subjecting 18 HD patients to a 12-week intradialytic bicycle ergometer exercise, found that aerobic exercise did not modulate the expression of NF-ĸB, Nrf2, or the Nrf2’s target gene NAD(P)H quinone oxidoreductase 1 (NQO1). However, none of these studies investigated the effects of resistance or aerobic exercise on cognitive function by modulating Nrf2 and NF-κΒ.

Furthermore, the brain-derived neurotrophic factor (BDNF), with its role in promoting neuronal survival, neurogenesis, synaptic plasticity, and cognitive function, is a particularly promising candidate for study in this area [112]. Previous studies have revealed that exercise-induced molecules, including osteocalcin, are vital in the upregulation of BDNF expression in the hippocampus, leading to enhanced cognitive functions, including mood, spatial memory, and learning [112]. Osteocalcin, a hormone specific to osteoblasts, is a key regulator of learning and memory through its modulation of BDNF. Importantly, higher levels of osteocalcin have been linked to improved executive function and cognitive performance in middle-aged and older individuals [113]. It is noteworthy that even a single bout of high-intensity exercise in sedentary older males can significantly boost the serum levels of the bioactive form of osteocalcin, known as uncarboxylated osteocalcin (UO) [114]. The IL-6 is the catalyst for inducing osteocalcin release in the bone and differentiation in UO, underlining its crucial role in this process. Low serum BDNF levels have been associated with depressive symptoms in HD patients, while high serum osteocalcin concentrations are considered an independent biomarker of osteoporosis in CKD patients. There is also evidence that aerobic exercise increases BDNF expression in older humans by regulating BDNF gene expression in the hippocampus. Based on previous research in mice models, exercise induces hippocampal BDNF through a peroxisome proliferator-activated receptor γ coactivator 1α (PGC-1α)/fibronectin type III domain-containing 5 (FNDC5) pathways. PGC-1α regulates neuronal FNDC5 gene expression, and PGC1a−/− mice show reduced FNDC5 expression in the brain. Forced expression of FNDC5 in primary cortical neurons increases BDNF expression, while RNAi-mediated knockdown of FNDC5 reduces BDNF [115]. Notably, a lack of PGC1-α has been linked to neurodegeneration [116,117], GABAergic dysfunction, and reduced parvalbumin expression in neurons [118]. Moreover, research shows that long-term forced treadmill running for three months can increase PGC1a expression in different brain parts [119]. Meanwhile, FNDC5 expression in the brain has been shown to correlate with exercise-induced improvements in brain function [115]. Studies have also shown that endurance and resistance exercise induces PGC1-α gene expression in the skeletal muscle of animal and older human models, supporting the important contribution of PGC-1α to the beneficial effects of exercise training at an advanced age by maintaining mitochondrial metabolic and antioxidant capacity [120,121]. Studies on CKD are only conducted on experimental mice, suggesting that exercise may be a possible indicator of PGC1-α adaptive response to stress in CKD patients [122]. However, the effects of exercise on BDNF levels in CKD patients need to be investigated. Additionally, whether resistance training can modulate BDNF in older healthy individuals or patients is unknown, regardless of the evidence showing that BDNF is correlated with higher handgrip strength [123,124].

Recent research also suggests that systematic exercise could counteract cognitive decline by triggering the release of neurotrophic factors such as insulin-like growth factor (IGF-1). Regarding the molecular mechanisms of IGF-1 in the brain, IGF-1 can cross the blood–brain barrier and enter the cerebrospinal fluid, performing several vital central nervous system functions, including neurogenesis and neuroprotection, through autocrine/paracrine or endocrine effects [125]. Human peripheral IGF-1 levels show a rapid increase in response to physical exercise [126]. This surge is a key factor in promoting exercise-induced neurogenesis and memory enhancement, making it a potential mediator in the cognitive benefits of exercise, alongside BDNF [127,128,129]. IGF-1 also contributes significantly to the exercise-induced effects of BDNF on recall [130]. In ESRD patients, lower levels of IGF-1 are associated with increased mortality. Recent research has also shown that cognitive functioning may be related to the GH-IGF-1-IGFBP3 axis [131]. Increased serum levels of IGF-1 might be linked to dementia pathologies, as IGF-1, with its potential to enhance amyloid-beta clearance from the brain, plays a crucial role in improving cognitive functions. In contrast, low levels of serum IGF-1 are a risk factor for dementia [33]. Prelevic et al. [132] showed that ESRD patients with lower scores in the mini mental state examination (MMSE), indicating severe cognitive impairment, had lower levels of IGF-1 and IGFBP3 compared to those with higher MMSE scores and no cognitive impairment. Interestingly, their study revealed that IGF-1 can be considered a novel biomarker for assessing cognitive functioning in CKD patients. Considering the correlation between exercise and IGF-1 levels with respect to cognitive frailty in CKD patients, studies in pre-dialysis CKD patients are few and controversial [133]. Studies on frail elders [134] show that resistance training increases the expression of IGF-1 in skeletal muscles, while, in HD patients [135], it leads to an improved IGF-1 status. Nindl et al. [136] showed that 12-week intradialytic resistance training in ESRD patients induced a decline in total IGF-I but did not alter the proportion of IGF-I circulating in free, ternary, or non-ternary molecular complexes. However, these exercise studies have yet to investigate the possible effects of exercise on IGF-1 levels in relation to cognitive function, even though the research mentioned above indicates a highly correlated relationship among exercise, cognitive impairment, and IGF-1.

## 5. Conclusions

In conclusion, the potential benefits of understanding the role of PGC1-α, BDNF, IGF-1, Nrf2, and NF-κB in exercise-induced adaptations for CKD patients’ cognitive frailty are promising. Future studies should further investigate the dynamics of their expression, signaling, and variation in levels. This understanding could open new avenues for treatment and management, offering hope for the future of CKD research.

## Figures and Tables

**Table 1:** Prevalence of cognitive frailty in patients with CKD.

Authors, Year	Design	Sample Size	Cognitive Function Tests	Prevalence of Cognitive Impairment
Kurella et al., 2004 [6]	Cross-sectional study	N = 160 N1 = 80 non-dialysis, CKD (stage III to IV) patients N2 = 80 ESRD undergoing HD	3MS, trails B, CVLT	A total of 27% in ESRD, 15% in patients with no advanced CKD and non in subjects with mild to moderate CKD.
Murray et al., 2006 [32]	Cross-sectional study	N = 338 ESRD patients un-dergoing HD	3MS, HVLT-R, 9 color trails 1 and 210 (a test similar to Halsted–Reitan trails A and B, but using alternating colors instead of numbers for trail B), Stroop interference test, BVMT-R, COWAT, clock-drawing test, Wechsler digit span, and the geriatric depression scale (short form)	A total of 13.9% were classified with mild impairment, 36.1% with moderate impairment, 37.3% with severe impairment, and 12.7% with normal cognition. Only 2.9% had a documented history of cognitive impairment.
Sehgal et al., 1997 [11]	Cross-sectional study	N = 336 ESRD patients undergoing HD	MMSE	A total of 22% had mild mental impairment and 8% moderate to severe mental impairment.
Cook et al., 2008 [30]	Cross-sectional study	N = 162 ESRD patients undergoing HD	MMSE	A total of 27% had cognitive impairment.
Tamura et al., 2010 [33]	Cross-sectional study	N = 383 ESRD patients undergoing HD	3MS impairment in executive function (trails B)	By using the 3MS, the cognitive impairment prevalence was 26% among HD patients.
Brady et al., 2009 [31]	Randomized controlled trial	N = 236 Veterans with ESRD	TICSm, digit span forward—span, digit span backward—span, verbal fluency, global cognition z-score composite, memory z-score composite, geriatric depression scale	The cognitive prevalence rate was 20% among participants.
Leinau et al., 2009 [29]	Observational cohort study	N = 109 ESRD patients undergoing HD	MMSE, EXIT25	Of the participants younger than 60 years old, 30% had cognitive impairment. In contrast, those older than 60 years had a higher cognitive impairment prevalence (48%).
Gela et al., 2021 [27]	Comparative Cross-Sectional Study	N = 116 CKD patients	SMMSE	The prevalence of cognitive impairment among CKD patients was 49.1%.
Viji et al., 2009 [28]	Observational and cross-sectional study	N = 210 CKD patients	MoCA	A total of 53.8% had mild cognitive impairment and 46.2% had normal cognitive status.
Williams et al., 2013 [26]	Observational and cross-sectional study	N = 79 CKD patients (Stage III to V)	CSI’D, TMTA, TMTB	More CKD patients had cognitive impairment compared with controls using CSI’D (51.9% versus 2.5%, *p* < 0.001), TMTA (53.2% versus 0%, *p* < 0.001), and TMTB (40% versus 0%, *p* < 0.001).

Note: CKD: chronic kidney disease; ESRD: end-stage renal disease; HD: hemodialysis; 3MS: modified mini mental state examination; Trails B: trailmaking test B; CVLT: California verbal learning trial; HVLT-R: Hopkins verbal learning test-revised; BVMT-R: brief visuospatial memory test revised; COWAT: controlled oral word association test; MMSE: mini mental state examination; TICSm: telephone interview for cognitive status—modified; EXIT25: 25-item executive interview; SMMSE: standardized mini mental state examination; MoCA: Montreal cognitive assessment questionnaire; CSI’D: community screening instrument for dementia; ΤΜΤA: trail making test A; ΤΜΤΒ: trail making test B.

**Table 2:** Studies examining the clinical efficacy of exercise with respect to cognitive frailty in patients with CKD.

Authors, Year	Design	Sample Size	Duration, Type of Exercise, and Groups	Cognitive Measurements	Functional Capacity and Other Measurements	Main Results after Exercise Training Programs
Kren et al., 2023 [94]	RCT	Ν = 44 HD patients	Intradialytic cycling and cognitive training 3 days per week for 12 weeks (exercise group) vs. standard care (control group).	MoCA, SDMT	HGS, 10-STS, stork balance test	Significant time * group interaction effect for SDMT (*p* < 0.001) and MoCA (*p* < 0.001). No significant interaction was observed for 10-STS, HGS, and the stork balance test (*p* > 0.05).
McAdams et al., 2018 [95]	RCT	N = 20 HD patients	Three months of intradialytic training (exercise group) vs. cognitive training (cognitive group) vs. standard care (control group).	3MS, TMTA, TMTB	-	Cognitive decline in psychomotor speed and executive function seen with standard care was possibly prevented by cognitive and exercise training, but not in all domains.
Manfredini et al. [96]	RCT	N = 227 HD patients	Six months normal physical activity (control; *n* = 145) vs. personalized walking exercise program at home (*n* = 151); 227 patients (exercise *n* = 104; control *n* = 123).	Cognitive function domain was assessed through KDQOL-SF	6MWT, 5xSTS, KDQOL-SF	After 6 months of training, HD patients improved their 6MWT and 5xSTS scores. The cognitive function score (*p* = 0.04) and quality of social interaction score (*p* = 0.01) in the kidney disease component of the KDQOL-SF improved significantly in the exercise group.
Nakamura-Taira et al., 2021 [97]	Quasi-cluster RCT	N = 42 HD patients	Six months, three times per week, intradialytic resistance intervention (exercise group) vs. stretching (control group) and 12-month follow-up.	MoCA-J	PHQ-9, AIS, NPI-Q, exercise self-efficacy	No significant effects on depression, cognitive function, and NPI-Q.
Belik et al., 2018 [98]	RCT	N = 30 HD patients	Four months intradialytic aerobic training (exercise group) vs. standard care (control group).	MMSE	IPAQ, Transcranial Doppler for cerebral blood flow assessment	Significant improvements of cognitive impairment and basilar maximum blood flow velocity in the exercise group.
Otobe et al., 2022 [99]	RCT	N = 44 Non-dialysis CKD patients	Group exercise training at the hospital once a week and independent exercise at home twice a week or more for 24 weeks (exercise group) vs. general care (control group).	Wechsler Memory Scale-Revised	-	Patients in the exercise group showed significantly greater changes in the Wechsler memory scale-revised logical memory delayed recall (*p* = 0.03) and in the immediate and delayed recall (*p* = 0.02) scores compared to patients in the control group.
Martins et al., 2011 [100]	RCT	N = 86 HD patients	Six months exercise (exercise group) vs. usual care (control group).	3MS	-	Better cognitive function was observed in active patients compared to the inactive ones (*p* < 0.05). Moreover, active patients over 60 years of age had better cognitive results than untrained ones (*p* < 0.05).

Note: *: and; HD: hemodialysis; CKD: chronic kidney disease; RCT: randomized clinical controlled trial; MoCA: Montreal cognitive assessment; SDMT: symbol digit modalities test; HGS: handgrip strength test; 10-STS: 10-repetition sit-to-stand test; 3MS: modified mini mental state exam; TMTA: trail making test A; TMTB: trail making test Β; 6MWT: 6-min walking test; 5xSTS: 5 times sit-to-stand test; KDQOL-SF: kidney disease quality of life short form; PHQ-9: patient health questionnaire-9: MoCA-J: Japanese version of the Montreal cognitive assessment; AIS: Athens insomnia scale; NPI-Q: neuropsychiatric inventory-brief questionnaire; IPAQ: international physical activity questionnaire; MMSE: mini mental state examination.

## Data Availability

The data presented in this study are available in the above tables.
